# An Innovative Artificial Intelligence–Based App for the Diagnosis of Gestational Diabetes Mellitus (GDM-AI): Development Study

**DOI:** 10.2196/21573

**Published:** 2020-09-15

**Authors:** Jiayi Shen, Jiebin Chen, Zequan Zheng, Jiabin Zheng, Zherui Liu, Jian Song, Sum Yi Wong, Xiaoling Wang, Mengqi Huang, Po-Han Fang, Bangsheng Jiang, Winghei Tsang, Zonglin He, Taoran Liu, Babatunde Akinwunmi, Chi Chiu Wang, Casper J P Zhang, Jian Huang, Wai-Kit Ming

**Affiliations:** 1 Department of Public Health and Preventive Medicine School of Medicine Jinan University Guangzhou China; 2 Sun Yat-sen University Cancer Center Guangzhou China; 3 College of Information Science and Technology Jinan University Guangzhou China; 4 International School Jinan University Guangzhou China; 5 School of International Studies Sun Yat-sen University Guangzhou China; 6 School of Journalism and Communication Jinan University Guangzhou China; 7 Faculty of Economics and Business University of Groningen Groningen Netherlands; 8 Department of Obstetrics and Gynecology Brigham and Women’s Hospital Boston, MA United States; 9 Center for Genomic Medicine Massachusetts General Hospital Harvard Medical School, Harvard University Boston, MA United States; 10 Department of Obstetrics & Gynaecology The Chinese University of Hong Kong Hong Kong Hong Kong; 11 School of Public Health The University of Hong Kong Hong Kong Hong Kong; 12 Multidisciplinary Collaborative Research Centre for Environment and Health, Department of Epidemiology and Biostatistics, School of Public Health St Mary’s Campus, Imperial College London London United Kingdom

**Keywords:** AI, application, disease diagnosis, maternal health care, artificial intelligence, app, women, rural, innovation, diabetes, gestational diabetes, diagnosis

## Abstract

**Background:**

Gestational diabetes mellitus (GDM) can cause adverse consequences to both mothers and their newborns. However, pregnant women living in low- and middle-income areas or countries often fail to receive early clinical interventions at local medical facilities due to restricted availability of GDM diagnosis. The outstanding performance of artificial intelligence (AI) in disease diagnosis in previous studies demonstrates its promising applications in GDM diagnosis.

**Objective:**

This study aims to investigate the implementation of a well-performing AI algorithm in GDM diagnosis in a setting, which requires fewer medical equipment and staff and to establish an app based on the AI algorithm. This study also explores possible progress if our app is widely used.

**Methods:**

An AI model that included 9 algorithms was trained on 12,304 pregnant outpatients with their consent who received a test for GDM in the obstetrics and gynecology department of the First Affiliated Hospital of Jinan University, a local hospital in South China, between November 2010 and October 2017. GDM was diagnosed according to American Diabetes Association (ADA) 2011 diagnostic criteria. Age and fasting blood glucose were chosen as critical parameters. For validation, we performed k-fold cross-validation (k=5) for the internal dataset and an external validation dataset that included 1655 cases from the Prince of Wales Hospital, the affiliated teaching hospital of the Chinese University of Hong Kong, a non-local hospital. Accuracy, sensitivity, and other criteria were calculated for each algorithm.

**Results:**

The areas under the receiver operating characteristic curve (AUROC) of external validation dataset for support vector machine (SVM), random forest, AdaBoost, k-nearest neighbors (kNN), naive Bayes (NB), decision tree, logistic regression (LR), eXtreme gradient boosting (XGBoost), and gradient boosting decision tree (GBDT) were 0.780, 0.657, 0.736, 0.669, 0.774, 0.614, 0.769, 0.742, and 0.757, respectively. SVM also retained high performance in other criteria. The specificity for SVM retained 100% in the external validation set with an accuracy of 88.7%.

**Conclusions:**

Our prospective and multicenter study is the first clinical study that supports the GDM diagnosis for pregnant women in resource-limited areas, using only fasting blood glucose value, patients’ age, and a smartphone connected to the internet. Our study proved that SVM can achieve accurate diagnosis with less operation cost and higher efficacy. Our study (referred to as GDM-AI study, ie, the study of AI-based diagnosis of GDM) also shows our app has a promising future in improving the quality of maternal health for pregnant women, precision medicine, and long-distance medical care. We recommend future work should expand the dataset scope and replicate the process to validate the performance of the AI algorithms.

## Introduction

Gestational diabetes mellitus (GDM), common in pregnancy, exerts negative effects on both mothers and their newborns, including cesarean delivery, shoulder dystocia, macrosomia, neonatal hypoglycemia, post-GDM type 2 diabetes mellitus, cardiovascular disease of pregnant women, and increased risk of obesity and type 2 diabetes mellitus on the offspring [1]. However, if GDM can be diagnosed at an early stage, early interventions can be implemented to maximally reduce its adverse consequences [2,3]. Although GDM prevalence in some developing African countries is high (eg, 8.2% in Nigeria and 9.5% in Tanzania) [4], pregnant women are less likely to receive adequate health care due to the lack of skilled health workers [5]. Other factors, such as poverty, inadequate medical services, long distance to hospitals, less access to information, and culture and traditions also prevent women from seeking care during pregnancy.

Artificial intelligence (AI) has been widely used in disease diagnosis in recent years [6,7]. Several advanced AI algorithms, such as deep learning, support vector machine (SVM), and convolutional neural network, have shown comparable performance to clinicians [8]. Major advanced AI approaches yield significant discriminative performance with relatively high sensitivity, specificity, and accuracy in object-identifying tasks [9,10]. At the same time, the world has witnessed the instantaneity of reporting and the consistency of producing results by AI [11]. AI is becoming more suitable for use in clinical daily practice [12] and offers the advantage of greater accuracy and efficiency [13]. An AI-driven dietary platform has been developed for diabetes management [14], and AI tools can enhance diabetes care for individuals and societal health [15,16].

Due to the advantages above, AI is expected to be further studied and implemented in the GDM diagnosis field to maximize social and economic benefits. A systematic review and meta-analysis on telemedicine technologies for diabetes in pregnancy conducted by our team in 2016 showed that telemedicine technologies can streamline clinical care delivery and improve maternal satisfaction [17]. We also evaluated the current state of GDM diagnosis programs by searching Scopus, Web of Science, PubMed, and Embase for studies published in English from inception up to November 17, 2019, using the keywords “gestational diabetes mellitus,” “GDM,” “GDM screening,” “GDM detection,” “GDM diagnosis,” “machine learning,” “artificial intelligence (AI),” and “deep learning.” Although some papers applied AI on screening or early diagnosis of GDM [18,19], they only used the expert system or risk score model instead of up-to-date AI algorithms such as random forest. Recently, a team from Israel applied top 20 contributing features such as baseline risk score and glucose challenge test results of previous pregnancy. A machine learning model based on national electronic health records reached high accuracy for GDM diagnosis [20]. Therefore, we intend to establish a GDM diagnosis tool using AI technology for women in low-resource areas. As our app targets to serve patients in resource-limited areas, it would be more practical and accessible if we can use only fasting glucose value and other patient’s basic health information such as age, body weight, and height. This study (referred to as GDM-AI study, ie, the study of AI-based diagnosis of GDM) aims to validate and rank the performance and applicability of AI algorithms in diagnosing GDM and to develop an innovative AI application for maternal health care. This paper will also present the ideas behind our app, as well as its contributions and prospects.

## Methods

### Recruitment

Our retrospective study initially involved 12,316 pregnant women who delivered a singleton at the First Affiliated Hospital of Jinan University (Guangzhou, Guangdong, China) from November 1, 2010, to October 31, 2017. We obtained ethics review and approval from the research ethics committee of the Jinan University. Medical records were used, and all data was confidential with anonymized numbers. The study excluded 12 pregnant women, as their profiles were not complete. A total of 12,304 pregnant women with full profile after admission were used as the development set and were diagnosed with or without GDM according to the International Association of Diabetes and Pregnancy Study Groups (IADPSG) diagnosis criteria. Patients were excluded if they met any of the following criteria: non-Chinese, multiple gestations, oral glucose tolerance test performed before 12 weeks, delivery in another hospital, major fetal malformation, or without patient clinical outcome in the electronic medical records. We extracted clinical data from the database of the First Affiliated Hospital of Jinan University (Guangzhou, Guangdong, China) as the development set, including the clinical baseline characteristics, maternal and neonatal complications, along with their clinical outcomes. The demographics and clinical characteristics of the patients are presented in Table 1. Another dataset of 1655 cases was obtained with the same criteria as an external validation set from Prince of Wales Hospital, the affiliated teaching hospital of the Chinese University of Hong Kong.

**Table 1:** Baseline characteristics.

Demographic and clinical variables	Developmental (Training) set (N=12,304)			External validation set (N=1655)		
	GDM^a^ (n=2761) (mean, SD)	NGT^b^ (n=9543) (mean, SD)	*P* value	GDM (n=240) (mean, SD)	NGT (n=1415) (mean, SD)	*P* value
Age (year)	30.21 (4.42)	28.50 (3.98)	<.001	32.87 (4.71)	30.33 (4.82)	<.001
Fasting Glucose Value (mmol/L)	4.89 (0.73)	4.37 (0.36)	<.001	4.74 (0.58)	4.32 (0.28)	<.001
1-h postload plasma glucose (mmol/L)	9.82 (1.84)	7.33 (1.38)	<.001	10.16 (1.63)	7.25 (1.35)	<.001
2-h postload plasma glucose (mmol/L)	8.53 (1.65)	6.47 (1.04)	<.001	8.63 (1.23)	6.24 (1.02)	<.001

^a^GDM: gestational diabetes mellitus

^b^NGT: normal glucose tolerance

### Study Design

All pregnant women received 2-hour 75 g oral glucose tolerance test according to the American Diabetes Association (ADA) 2011 criteria. As per the ADA 2011 diagnostic criteria for GDM, the upper limits of the blood glucose for fasting, 1-hour postprandial, and 2-hour postprandial blood glucose are 5.1 mmol/L, 10.0 mmol/L, and 8.5 mmol/L, respectively. Those with one or more abnormal value(s) will be diagnosed as GDM.

Each case was carefully reviewed by 2 experts individually. The patient’s evaluation result, which would be finished within a week, was assigned to GDM cohort and non-GDM cohort, depending on the laboratory data change, clinical manifestation, clinical intervention, and final diagnosis. If a discrepancy occurred, the case was reviewed by another third expert and labeled after consensus was reached. In the developmental dataset, cases were labeled as either normal or GDM, according to the ADA 2011 criteria.

We compared baseline characteristics between the 2 cohorts (nonGDM and GDM) as shown in Table 1 and found that there were significant differences between them, except height. Therefore, we tested several times different combinations of baseline characteristics in AI algorithms to get the best combination for distinguishing GDM from nonGDM. Eventually, we came to a combination of age and fasting blood glucose.

We tested 9 advanced AI algorithms, including SVM, random forest, AdaBoost, k-nearest neighbors (kNN), naive Bayes (NB), decision tree, logistic regression (LR), eXtreme gradient boosting (XGBoost), and gradient boosting decision tree (GBDT), by utilizing age and fasting blood glucose in the collected datasets. We used the development set for model development and carried out internal validation with 5-fold cross-validation. The development set was randomly split into 5 folds. Within each fold, we used 1 fold for validation and the rest for training the model. Results were calculated by averaging the results from the 5 separate experiments.

GDM classification performances of the trained models were validated using the internal validation with 5-fold cross-validation and the external validation set, which was evaluated using the area under the receiver operating characteristic curve (AUROC).

### Statistical Analysis

We hypothesized that the AI model is at least comparable to the ADA 2011 criteria. Thus, we compared performances of the advanced AI models to ADA 2011 diagnosis results. Accuracy, sensitivity, specificity, area under the curve (AUC), positive predictive value (PPV), negative predictive value (NPV), Brier score, positive likelihood ratio, and negative likelihood ratio were calculated for each algorithm.

For the validation datasets, performance was evaluated from the probability values by using the AUROC curve analysis for GDM detection with python 3.6.8 based on Jupyter Notebook (Project Jupyter).

Performance evaluation was achieved via receiver operating characteristic (ROC) curve analysis, and calculation of AUC using the “pROC” package. The cut-off value (0.501) for the model was determined by the “OptimalCutpoints” package using the Youden method. Asymptotic 2-sided 95% CIs were computed for the logit transform of each proportion (ie, sensitivity and specificity). All analyses were performed using Stata (version 14.0).

## Results

A total of 12,304 outpatient cases were included in the development dataset. Among these women, 77.6% (9543/12304) were non-GDM as per the ADA 2011 criteria, and 22.4% (2761/12,304) women were diagnosed as GDM. GDM was found in 14.5% (240/1655) cases among the external validation dataset.

A 5-fold cross-validation was applied for the internal dataset, and accuracy and false positives were recorded. All the evaluation indices are presented in Table 2 and Table 3.

**Figure 1:**
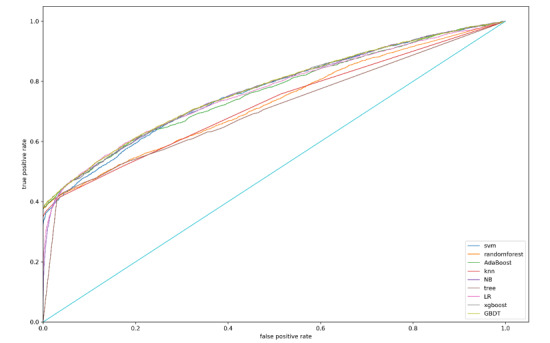
Overall area under the receiver operating characteristic curves for internal validation dataset. SVM: support vector machine; knn: k-nearest neighbors; NB: naive Bayes; LR: logistic regression; XGBoost: eXtreme gradient boosting; GBDT: gradient boosting decision tree.

ROC curves are shown in Figure 1, which indicates the higher performance of AUC of SVM, AdaBoost, NB, LR, XGBoost, and GBDT.

For the internal validation dataset, the best performance of AUC was for GBDT and XGBoost, which had relatively higher accuracy, specificity, and PPV, while their Brier scores were lower than those of others (Table 2).

For the external validation dataset, the AUC of SVM for GDM was 0.78, with 88.7% accuracy and 100% specificity (Table 3). The specificity of NB was 98.2% for diagnosing GDM with AUC of 0.774 (Table 3). Demonstration of AI Application is shown in Table 4.

**Table 2:** The detection performance of 9 algorithms for the internal validation dataset.

Algorithms	Accuracy	Sensitivity	Specificity	PPV^a^	NPV^b^	Brier score	AUC^c^
SVM^d^	0.849	0.377	0.985	0.880	0.845	0.151	0.766
Random forest	0.833	0.432	0.949	0.709	0.852	0.167	0.728
AdaBoost	0.860	0.376	1	1	0.847	0.140	0.763
kNN^e^	0.841	0.415	0.964	0.768	0.851	0.159	0.723
NB^f^	0.845	0.367	0.983	0.860	0.843	0.155	0.768
Decision tree	0.838	0.431	0.956	0.738	0.853	0.162	0.706
LR^g^	0.844	0.363	0.984	0.865	0.842	0.156	0.765
XGBoost^h^	0.860	0.377	1	1	0.847	0.140	0.771
GBDT^i^	0.860	0.376	1	1	0.847	0.140	0.772

^a^PPV: positive predictive value.

^b^NPV: negative predictive value.

^c^AUC: area under the curve.

^d^SVM: support vector machine.

^e^kNN: k-nearest neighbors.

^f^NB: naive Bayes.

^g^LR: logistic regression.

^h^XGBoost: eXtreme gradient boosting.

^i^GBDT: gradient boosting decision tree.

**Table 3:** The detection performance of 9 algorithms for the external validation dataset.

Algorithms	Accuracy	Sensitivity	Specificity	PPV^a^	NPV^b^	Brier score	AUC^c^
SVM^d^	0.887	0.221	1	1	0.883	0.113	0.780
Random forest	0.838	0.263	0.936	0.409	0.882	0.162	0.655
AdaBoost	0.882	0.183	1	1	0.878	0.118	0.736
kNN^e^	0.862	0.254	0.965	0.550	0.884	0.138	0.669
NB^f^	0.878	0.263	0.982	0.716	0.887	0.122	0.774
Decision tree	0.841	0.242	0.942	0.414	0.880	0.159	0.614
LR^g^	0.877	0.258	0.983	0.713	0.887	0.123	0.769
XGBoost^h^	0.882	0.183	1	1	0.878	0.118	0.742
GBDT^i^	0.882	0.183	1	1	0.878	0.118	0.757

^a^PPV: positive predictive value.

^b^NPV: negative predictive value.

^c^AUC: area under the curve.

^d^SVM: support vector machine.

^e^kNN: k-nearest neighbors.

^f^NB: naive Bayes.

^g^LR: logistic regression.

^h^XGBoost: eXtreme gradient boosting.

^i^GBDT: gradient boosting decision tree.

**Table 4:** Demonstration of AI application.

Sample	Age	Fasting glucose (mmol/L)	Result with AI application
1	35	5.2	GDM^a^
2	25	4.5	No GDM
3	27	4.8	No GDM
4	33	3.5	No GDM
5	37	5.6	GDM
6	30	4.3	No GDM
7	30	6.7	GDM
8	27	5.4	GDM

^a^GDM: gestational diabetes mellitus.

The goal of this preliminary research was to develop an app and demonstrate the soundness of the methods. The users can put data into the app, which will be transferred simultaneously to the doctors and other medical personnel. In this way, the app provides a platform for the doctor to get informed of the users’ health status and give intervention to the GMD patients, while the app user can keep track of their physical conditions.

To be more specific, our mobile app is expected to quantify the daily lives of pregnant women and optimize diet, exercise, and sleep to help them maximize their well-being. The app serves as an “online intelligent nurse” that can answer simple questions to reduce obstetric doctors’ workload. The built-in model was chosen for our app, which will still be tested, adjusted, and improved continually. There is a need to collect data regarding pregnant women’s daily habits and body conditions and to use machine learning algorithms to study these data. Such data will also be sent to a cloud database and analyzed again to determine the relationship between the amount of exercise and caloric intake to help users balance exercise and diet. Moreover, it will give a prediction of the probability of users having other diseases and their next-day blood glucose levels. The actual blood glucose uploaded by the user will be used to make comparisons with the predicted value to verify, correct, or improve our model.

**Figure 2:**
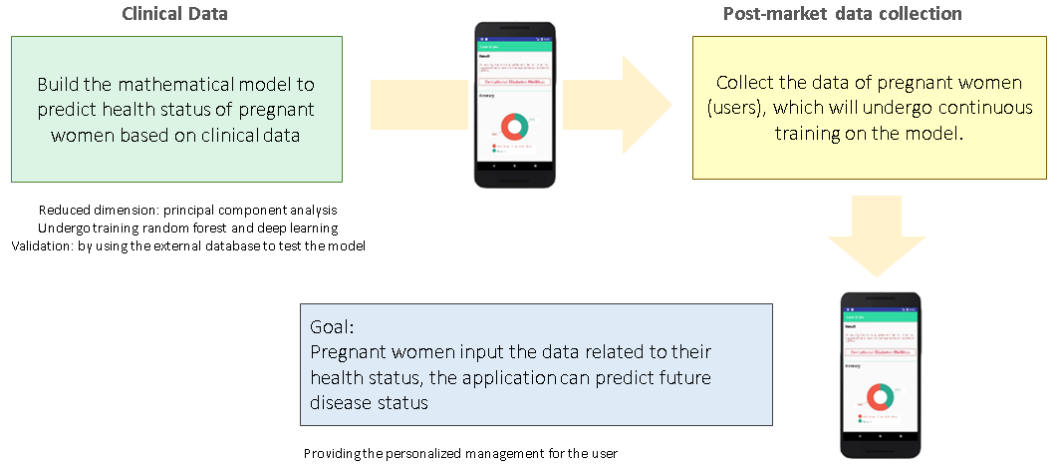
How the AI app works.

**Figure 3:**
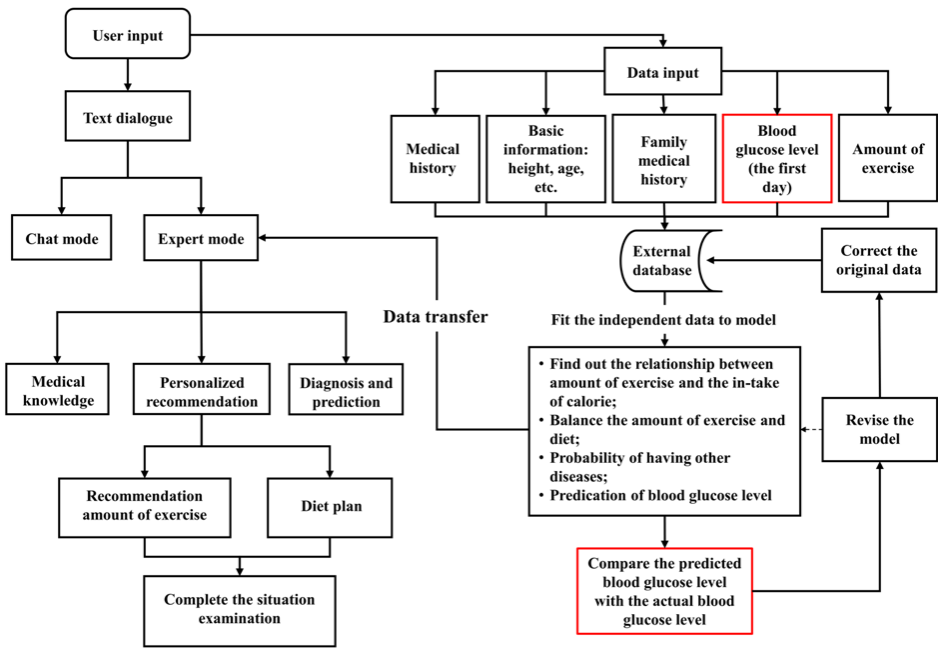
Structure of the app.

The detailed process of app development is illustrated in Figure 2 and Figure 3.

**Figure 4:**
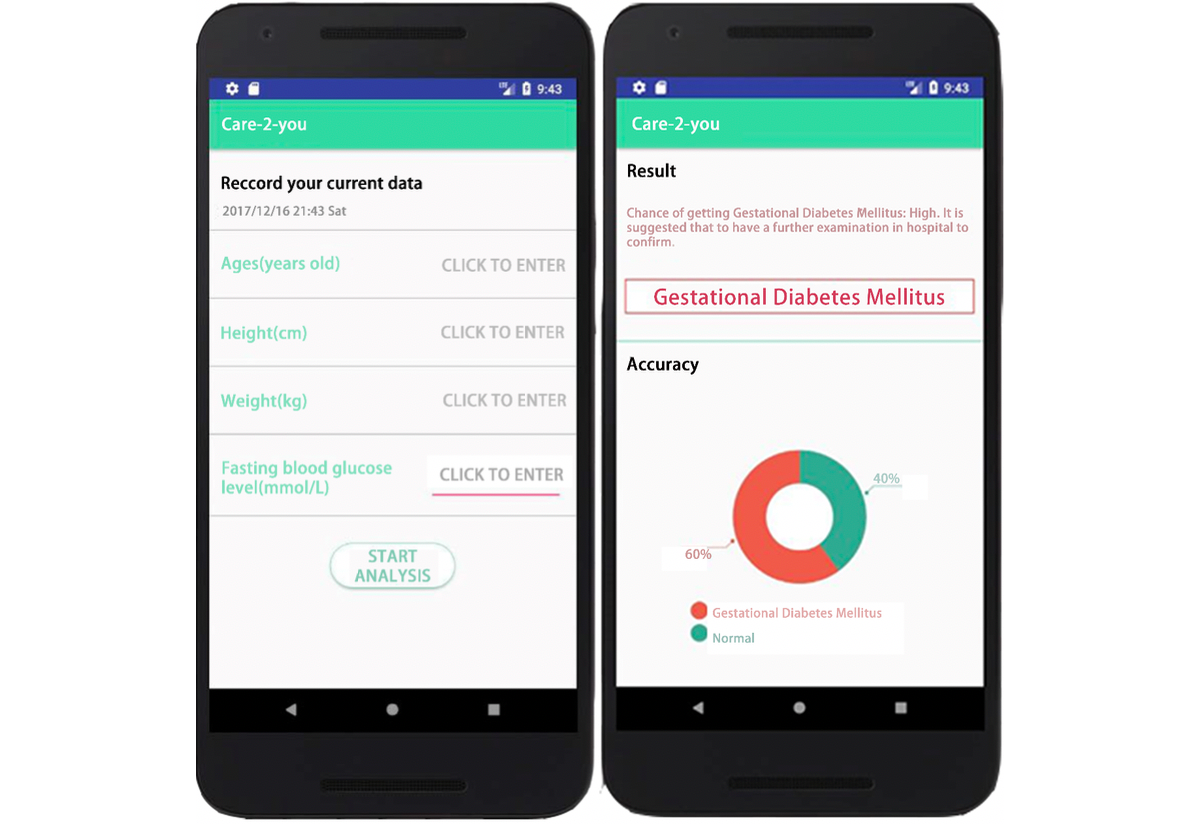
Interface of the app.

The app interface is shown in Figure 4.

## Discussion

### Principal Results

In this study, we described the use of 9 AI algorithms for the diagnosis of GDM, utilizing only the age and fasting blood glucose. Internal and external validation demonstrated that AI algorithms provide strong or moderate evidence to rule in diagnosing GDM under the same medical resource conditions. Moreover, SVM retained high performance in accuracy, specificity, PPV, positive likelihood ratio, and AUROC for achieving the correct diagnosis, which suggests the potential of the SVM algorithm to make highly accurate diagnosis decisions. NB also provides moderate evidence to rule in diagnosing GDM under the same medical resource conditions.

Established diagnostic tools such as ADA 2011 criteria have been used to decide whether or not a pregnant woman has GDM. However, ADA 2011 criteria are resource-demanding, and thus may not be utilized in undeveloped areas in China. Moreover, these diagnostic tests are both expensive and difficult for pregnant women given their body conditions during pregnancy. Compared with those established diagnostic tools in the ADA 2011 criteria, the automatic diagnosis algorithm can provide a real-time and accurate diagnosis with fewer medical resources. Also, such algorithm-based diagnosis would be less expensive since it requires fewer equipment and professional medical staff. As GDM puts a major economic burden on the public health care system, the government and health policymakers can evaluate the economic benefits of our free app (which can inform patients of their diagnoses and facilitate early medical intervention, if needed) based on the results of this study and seek international cooperation [21-26].

We believe that the future focus of AI in medicine should be directed towards solving medical problems in resource-insufficient areas, and this application will likely help address the shortage of medical resources. In addition to GDM, we believe that AI could be applied to other diseases.

### Limitations

First, the development set all comes from one hospital, but the external validation set has fixed this problem. Second, the data is retrospective and therefore, not up to date. Third, the datasets pertain to the Guangdong and Hong Kong populations, both in regard to patient and system characteristics. Applicability of our findings to other populations with distinct health care systems may need further investigations. Fourth, there is no algorithm that performs well in sensitivity compared with human experts, illustrating that the capability of AI-diagnosing GDM requires improvement.

Since the scope of our dataset is relatively small, the next step of our study will be to expand our internal dataset and repeat the process to validate that SVM performs well in different datasets. Databases from different jurisdictions can be included in our test. Although SVM outperformed in overall criteria, NB outperformed SVM according to Brier Score in both datasets. Therefore, a further investigation into the differences between SVM and NB will be carried out.

### Comparison With Existing Literature

Diagnosis for initial GDM is performed to aid further observation of pregnant women and to guide interventions. Several tools have been previously developed for diagnosing and predicting diabetes mellitus. However, these tools require detailed information of a pregnant woman, for example, all important factors regulating the patient’s blood glucose level [27], demographic information [18], or various blood glucose values [28]. Therefore, they are not suitable for pregnant women living in resource-limited rural areas. In contrast, our diagnosis mechanism only requires the fasting glucose value and the patient’s age, which enhances its application.

### Contribution of the Application

A core feature of the app is intelligent medical care. The process of collecting, extracting, processing, and presenting data and renewing the app are both automated. Therefore, it can save a lot of time and effort while performing at high efficacy and accuracy.

Moreover, if our app can be interconnected with wearable devices, the app can monitor, in real-time, the heart rates and blood pressure levels of pregnant women to inform them of their physical conditions with data and illustrations, such as whether they should continue to exercise or rest. Such intelligent health care will be beneficial for mothers-to-be in rural areas where medical resources might be in short supply, reassuring both pregnant women and their families.

Precision medicine is a modern branch of medicine that can give individualized medical care according to a patient’s genetic, biomarker, or psychosocial characteristics. Precision medicine remains expensive and difficult to deliver for most pregnant women in rural areas. However, our app provides the possibility for affordable precision medicine providers since it can respond with medical advice and individualized arrangements of daily exercises and diets.

The application also makes the long-distance medicine possible for those pregnant women living in rural areas, where medical services are insufficient and transportation is underdeveloped. Doctors can track the patient’s glucose level and other health information through the application in real-time and give diagnosis and suggestions through real-time online communication. The application can also be used for urban pregnant women who are too busy to attend time-consuming examinations.

In addition, through the realization of precision medicine and long-distance medical care, this app can use available obstetric resources to detect a relationship between health data and pregnant women’s health to search for new ways to manage risks during pregnancy and to provide more effective management of GDM. In this way, our app can improve the efficiency and quality of maternal health care, particularly in rural areas, and help revitalize the current global medical system.

The AI-based app can promote long-distance medical care by making timely and accurate diagnosis in low-resource conditions while possibly lowering the cost of GDM diagnosis and improving the quality and efficiency of maternal health care in rural areas. First, the introduction of this application will effectively be improving diagnostic rate of gestational diabetes in low-resource areas and thus preventing high risks in pregnancies. Second, AI makes intervention for high-risk women possible. Third, AI application might be complementary solutions to reduce diagnostic delay and delay of getting prevention advice and possible treatment in underserved rural GDM population globally.

### Conclusion

There are many challenges associated with inadequate obstetric services in rural areas around the world, yet this also provides an opportunity for the development of AI in the medical field. In our study, 9 algorithms (SVM, random forest, AdaBoost, kNN, NB, decision tree, LR, XGBoost, and GBDT) were tested to identify the best-performing algorithm in the diagnosis of GDM. SVM performed best and was adopted to develop a mobile app. Although further experiments are needed, we believe the developed app will promote precision medicine and long-distance medical care while improving the quality and efficiency of maternal health care in rural areas.

## Abbreviations

ADA: American Diabetes Association
AI: artificial intelligence
AUC: area under the curve
AUROC: areas under the receiver operating characteristic
GBDT: gradient boosting decision tree
GDM: gestational diabetes mellitus
GDM-AI: AI-based diagnosis of GDM
IADPSG: International Association of Diabetes and Pregnancy Study Group
kNN: k-nearest neighbors
LR: logistic regression
NB: naïve Bayes
NPV: negative predictive value
PPV: positive predictive value.
ROC: receiver operating characteristic
SVM: support vector machine
XGBoost: eXtreme gradient boosting
